# Fall-Related Activity Avoidance among Persons with Late Effects of Polio and Its Influence on Daily Life: A Mixed-Methods Study

**DOI:** 10.3390/ijerph18137202

**Published:** 2021-07-05

**Authors:** Christina Brogårdh, Jan Lexell, Catharina Sjödahl Hammarlund

**Affiliations:** 1 Department of Health Sciences, Lund University, 221 00 Lund, Sweden; jan.lexell@med.lu.se (J.L.); catharina.sjodahl_hammarlund@med.lu.se (C.S.H.); 2 Department of Neurology, Rehabilitation Medicine, Memory Disorders and Geriatrics, Skåne University Hospital, 222 41 Lund, Sweden; 3 The PRO-CARE Group, School of Health and Society, Kristianstad University, 291 39 Kristianstad, Sweden

**Keywords:** activities of daily living, fear of falling, postpoliomyelitis syndrome, qualitative research

## Abstract

Falls are common among persons with late effects of polio (LEoP), which may lead to fear of falling and activity avoidance in everyday life. Here, we assessed the occurrence of fall-related activity avoidance among persons with LEoP and explored how these experiences influenced daily life. Fourteen ambulatory persons (seven women; mean age 70 years) with LEoP participated. They responded to the modified Survey of Activities and Fear of Falling in the Elderly (mSAFFE) and participated in individual interviews, which were analysed by systematic text condensation. Each quotation was deductively analysed from its representation with regard to mSAFFE. We found that many persons often avoided activities related to standing and walking, for example, taking a bath, performing household chores, walking outdoors, attending social events if there were stairs in the building and travelling by public transport, due to fear of falling, increased pain and fatigability. To facilitate the performance of daily activities participants expressed that strategic thinking and aids were important to use. In conclusion, fall-related activity avoidance is common in persons with LEoP, which negatively influence daily life and social participation. To increase daily functioning in this population, fall-related activity avoidance should be included in a multifaceted fall management program.

## 1. Introduction

Many persons who acquired polio in their childhood or youth experience new or increased impairments decades after their initial infection, so-called post-polio syndrome or late effects of polio (LEoP) [1]. Common impairments in persons with LEoP are muscle weakness, muscle fatigue, general fatigue and musculoskeletal pain during everyday activities and physical activities [2,3,4,5]. The underlying causes of LEoP are not entirely clear, but it is generally agreed that the new impairments occur due to a distal degeneration of axons in the enlarged motor units that developed during the recovery of the acute paralytic polio. At the age of around 50 years, people start to experience the “normal” age-related loss of motor neurons leading to many of the neuromuscular impairments, and this process seems to be accelerated in persons with LEoP [1]. Thanks to global vaccination programmes, which started in the 1950s, only a few countries in Africa and Asia today report new cases of polio, which in total amount to less than one hundred per year.

As approximately 60% of the persons that were affected by polio in their childhood develop LEoP, it is one of the most common motor neuron diseases in the world [1]. The new or increased impairments that many persons with LEoP report can lead to reduced balance and walking limitations [6], which increase the risk of falls [7,8,9,10]. Studies have shown that falls are very common among persons with LEoP, and 50% to 84% experience one or more falls per year [7,8,9,11,12,13]. In particular, falls seem to occur in the afternoon [7,8] and during activities related to walking [8,9]. Many persons with LEoP become injured when they fall, and even though bruises are most commonly reported, fractures and head injuries also occur [7,8,12,13,14]. The experiences of falls can lead to fear of falling [8,10,11,15], which in turn, may lead to worries and distress [16] and behavioural changes such as physical inactivity [3], and activity avoidance in daily life [16].

One rating scale that can be used to assess fall-related activity avoidance in daily life is the modified Survey of Activities and Fear of Falling in the Elderly (mSAFFE) [17]. The scale has previously been used among older adults [17] and in persons with Parkinson’s disease (PD) [18,19] to assess if various physical activities and social activities are avoided due to the fear of falling. However, even though the mSAFFE could be a valuable tool to assess fall-related activity avoidance in daily life, the drawback is that it comprises predefined questions, which may limit a deeper understanding of the phenomenon. Qualitative interviews could, therefore, be a valuable complement and add a more in-depth understanding of a person’s experience.

Although falls and fear of falling are common among persons with LEoP [7,8,9], no study has, to the best of our knowledge, described fall-related activity avoidance in this population and how it influences daily life. Such knowledge could aid clinicians in their planning of individualized rehabilitations interventions in order to improve overall functioning. Thus, the aim of this study was to assess the occurrence of fall-related activity avoidance in persons with LEoP and to explore how these experiences influence daily life.

## 2. Materials and Methods

### 2.1. Research Design

This study has a mixed-methods design [20] and is part of a larger qualitative project on experiences and consequences of ageing and falls among persons with LEoP [5,16]. Before the interviews, information about the project and various rating scales regarding self-reported impairments, walking limitations, fear of falling and fall-related activity avoidance were mailed to the participants to be filled in at home. In the present study, only data on fall-related activity avoidance are presented.

The advantage of using a mixed-methods design and combining predefined questions with participants’ experiences is that quantitative and qualitative data can be triangulated, enabling a broader and more in-depth understanding of a phenomenon [20], such as the influence of fall-related activity avoidance on daily life. 

### 2.2. Participants

From a post-polio rehabilitation clinic in southern Sweden, participants with a confirmed history of acute poliomyelitis in their childhood and with new impairments after a period of functional stability were recruited. All of them had previously taken part in a cross-sectional study of falls and fear of falling [8]. By strategically selecting participants from that cohort [8], a variation of participants’ characteristics, i.e., gender, age, functional level, years with LEoP, fall frequency and fear of falling, were obtained.

A total of 18 persons were contacted by telephone and informed about the project, and 14 agreed to participate (Table 1). The participants were on average 70 years, their mean age at the acute poliomyelitis infection was 4 years, and the mean duration of LEoP related impairments was 26 years. Most of them lived with a partner in their own home. A majority could walk at least 100 m and used a mobility device during ambulation. On average, they were highly concerned about falling (mean 33 points on the Falls Efficacy Scale—International, FES-I) [21,22,23]. 

### 2.3. Ethics

Prior to inclusion, all participants received oral and written information about the study and gave their informed consent to participate. The study was approved by the Regional Ethical Review Board, Lund, Sweden (Dnr: 2014/186), and the principles of the Declaration of Helsinki were followed.

### 2.4. Data Collection

The mSAFFE was used to assess fall-related activity avoidance in daily life [17]. The scale originates from the survey of activities and fear of falling in the elderly (SAFE) [24], but has somewhat fewer items as those with poor discriminant validity were omitted in the modified version [17]. Hence, the mSAFFE [17] includes 17 activities regarding indoor and outdoor walking, personal activities, household activities, attending social events, visiting a friend or relative and travelling by public transport. Each item has three response categories: 1 = never (avoid), 2 = sometimes (avoid) and 3 = always (avoid). The total mSAFFE score ranges from 17 to 51, and higher scores indicate more fall-related activity avoidance. The participants responded to the mSAFFE at home, which took about 15 min to complete, and brought their ratings to the post-polio rehabilitation clinic in connection with the interviews.

The individual semi-structured interviews were performed at the post-polio rehabilitation clinic by the first author (CB, physiotherapist) or the last author (CSH, physiotherapist and psychologist). The interviews were based on an interview guide with open-ended questions regarding experiences of ageing and falling among persons with LEoP, the consequences of falls, how these experiences influenced daily life and strategies to overcome hazardous situations and adversities. Follow-up questions such as: “Can you elaborate on that, please?” and “Please, describe.” were used. All interviews were audio recorded and transcribed verbatim. The interviewers had professional experience of persons with LEoP but had no clinical relation with the participants. The interviews lasted between 60 and 90 min.

### 2.5. Data Analysis

The qualitative analysis from the interviews was conducted before the analysis of mSAFFE in order to avoid potential bias. The interviews were analysed by systematic text condensation [25], which is a descriptive and explorative method inspired by phenomenological theory. First, the transcripts were read to obtain a general impression of the data and to identify and categorise preliminary themes. Next, meaning units were formulated into codes that represented the essence of the statements. During this phase, the first and last authors worked individually to uncover various perspectives and nuances of the data. The coded data were then organised by their conceptual representation into subcategories, and duplicates were removed. The subcategories were organised into categories, and the re-contextualised data were expressed as descriptions of the meaning of each category. Finally, representative quotes were selected for each category/subcategory. When designing and conducting the study, the COREQ checklist was followed [26].

The coded data were then reanalysed, and each quotation was deductively analysed from its representation with regard to fall-related activity avoidance. Each quotation that corresponded to an item in mSAFFE received the matching item number. The deductive analysis was done before the analysis of the results of mSAFFE. All authors (CB, CSH and JL, a physician with extensive experience of persons with LEoP) were involved in the interpretation of the results.

For the mSAFFE, descriptive statistics, i.e., number and percent (%), were used for the response categories of each items.

## 3. Results

### 3.1. The Ratings of Fall-Related Activity Avoidance According to mSAFFE

The activity that most participants (57%) always avoided was going out when it was slippery. More than two-thirds sometimes avoided or always avoided going for a walk, walking more than one kilometre or climbing stairs. More than half (>50%) of the participants reported that they sometimes or always avoided travelling by public transport, walking in a crowded place such as a store/shop, taking a bath and reaching for something above their head. On the other hand, activities that most of the participants (>70%) never avoided were going to the doctor or dentist, walking around indoors, preparing simple meals, bending down to pick up something and visiting a friend or a relative (Table 2).

### 3.2. How the Fall-Related Activity Avoidance Influenced Daily Life

From the deductive analysis, eight categories emerged. In Table 3, an overview of the categories and their relation to the various items in mSAFFE are presented.

#### 3.2.1. Going out When It Was Slippery Was Often Avoided Due to Fear of Falling

The disability following LEoP affected the ability to walk. Especially, outdoor walking when it was slippery on the ground or on the pavements was often avoided due to the fear of falling (cf. item 8). Many of the participants had fallen outdoors when it was slippery, and when the ground was frozen, only a thin layer of ice or snow on the ground was found treacherous enough to avoid going outside. For some participants, it meant that they could not go out during wintertime at all or had to wait until the ground was sanded.

*No… going out on the streets when it’s slippery, I don’t do that. And when, not just when it’s frozen but if there’s snow and no sand has been spread on the ground, I don’t do that.* (P 9)

However, some participants had developed strategies that allowed them to walk more safely outdoors and to reduce their fear of falling. Although they did not dare to walk very far, one strategy was to wear special winter shoes with a solid anti-slip sole. Another strategy was to use mobility aids, such as a rollator, cane or Nordic walkers. Some had also learnt to be extra cautious and to watch every step whilst walking on slippery surfaces.

*I put studs on my crutch, anti-slip soles. Then you need a good pair of winter shoes. You can’t walk outside with shoes with plastic soles. You have to make sure to wear good shoes when it’s slippery. You’re also extra careful. I don’t walk very far, just to the car.* (p. 2)

#### 3.2.2. Challenging to Walk Longer Distances Because of Increased LEoP-Related Impairments

Almost half of the participants avoided walking longer distances (i.e., more than 1 km, item 13), whereas going for a shorter walk was more seldom avoided (cf. item 7). The participants described that they had become more afraid of walking long distances and gradually felt more muscle fatigue and pain already after a couple of hundred meters. The reduced walking capacity when ageing with LEoP affected their ability to walk with friends and family members, as it was often too difficult to keep up with their speed.

*It’s probably the fatigue and the fact that I’ve become more afraid of walking. If I’m walking outside I can’t walk in the same way as those who I’m walking with. I need to be a bit more careful and walk at my own pace.* (p. 4)

*…to walk long distances… I avoid that… I notice, as I said, already after a couple of hundred meters that my leg gets really tired, then it becomes worse and worse the longer you walk.* (p. 11)

While some participants avoided going for a walk, others described that they became more alert afterwards if they had chosen a distance that corresponded to how they felt that day.

*We have several [walking rounds] at home… and we went for the long one today, “it feels good, let’s go for the long one today”… and it takes forty-five minutes or something like that. It feels really good when you get home… it makes me more alert.* (p. 9)

#### 3.2.3. Mobility Aids Facilitated Going to the Store and Other Crowded Places

Most of the participants reported that they never or sometimes avoided walking to the store and other crowded places (cf. items 1 and 10). However, the difficulty to move, as a consequence of ageing with LEoP, and the feeling of fatigability and instability when walking made it somewhat harder to go shopping and to carry home groceries. One thing that could facilitate shopping and social participation was to support oneself using a shopping cart or to take a powered wheelchair.

*If someone wants to go to a mall or walk and look around, then I take my powered wheelchair with me. I could wear them all out. Then I’m also participating with the others.* (p. 2)

#### 3.2.4. Walking Indoors Was Rarely Avoided but Could Still Be Very Challenging

Even though very few participants avoided to walk indoors (cf. item 12), they described several challenges and how to manage them. To be able to walk safely indoors and to reduce the risk of falling they often used support from walking aids, furniture or handles but also needed completely flat surfaces. Even small unevenness on carpets could be very risky. Additionally, doing two things at the same time, for example, walking and carrying a tray, was very challenging and, therefore, often avoided.

*… and carrying trays… for some strange reason you get more unstable with a tray in your hands. I don’t understand why, but it’s the thing of not falling when you have all the stuff on the tray… you tremble so much. Strange, but that’s how it is. So THAT I do avoid.* (p. 7)

#### 3.2.5. Household Chores Could Be Physically Demanding and Cause Strain on the Body

Another indoor activity that about one third of the participants always avoided was cleaning the house (cf. item 2). Vacuuming was one of the more physically demanding household chores, which added strain on the muscles and ligaments in the back and legs. Those who felt muscular fatigue and pain avoided such undertakings, but some could still manage by using various compensatory strategies, for example, sitting down whilst vacuuming.

*I don’t vacuum and those things anymore, I let my husband take care of that. I avoid that. It’s not good for me. My legs and my back start hurting a lot. I don’t know, I think it’s the position and all the turning around that does it. Vacuuming doesn’t make for a good ergonomic position. So, I don’t do that anymore…* (p. 7)

In order to be able to prepare simple meals (cf. item 3), it was necessary for many to do preparations and to plan how to perform the activity. For some participants, it was exhausting and painful to stand up whilst preparing meals. They perceived the back and legs to be too activated in a standing position, but by taking regular breaks and sitting down on a chair, they could still succeed in preparing and cooking for themselves and their family.

*I can manage standing up for a while, but then I have to rest every now and then. But it works, I shouldn’t complain. I’ve had a chair that I use in the kitchen, which I can move around with.* (p. 5)

Doing different household chores often required having the ability to reach to the upper shelves and to keep the balance, which were perceived as difficult by many. Nearly half of the participants sometimes avoided activities that required reaching for something above their head (cf. item 17). The muscle weakness in their lower limbs and the risk of falling meant that some did no longer dare to climb on a stool to reach something. However, by using various strategies and tricks, they could sometimes manage.

*… getting up on a stool and such. I’ve done that before, but now I’ve bought myself a little stepladder… getting up on a chair is a very big project for me... It’s not easy since I don’t have the power to [push away]… so I avoid it nowadays… Otherwise, I’ve thought of a trick for when I need to get something from a cabinet... I’ll take another thing and [use it to] push forward the thing I need until I can reach it.* (p. 7)

For some, bending down to pick up something (cf. item 14) from the lower shelves, from the floor or to pull weeds in the garden could also be tricky. Standing and leaning forward was strenuous, but taking regular breaks or sitting down made it possible to still do the gardening.

*…when you work in the garden, you can’t bend down too much because of the back. So, I take a thick foam rubber pad, put that in a plastic bag and sit instead…* (p. 9)

#### 3.2.6. Taking a Shower Was Seldom Avoided but Was Carried out with Precautionary Measures

Most of the participants said that they never avoided taking a shower (cf. item 6), whereas half of them expressed that they sometimes avoided taking a bath, as it could be challenging to climb in and out of the bathtub (cf item 5). Many avoided standing up when they showered to reduce the risk of falling. Some of the participants had previously experienced falls in the bathroom and, therefore, used various aids, such as banisters, shower chairs and non-slip mats, to increase safety. With support and developed strategies, for example, to be cautious and carefully plan how they should proceed in every moment, they could manage personal activities more easily.

*I feel scared when standing in the bathtub even if I’m on a rubber mat… I also have a handle by the tub and a sitting board….* (p. 3)

*If I’m taking a shower, I have to think about how to go about it. I have to hold onto that, sit on the bath stool. I don’t dare to stand, I’ve fallen before and it almost ended in disaster. You have to learn that you have to think at all times. And then things go well.* (p. 10)

#### 3.2.7. Attending Social Events Could Be Problematic If There Were Stairs in the Building

Attending a social event, for example, going to a sports facility, the theatre or visiting a friend, was perceived as important and, therefore, seldom avoided (cf. items 9 and 16). However, attending an event could be tough if there were stairs in the building (cf. item 11). The participants told us that they often managed a few steps, but walking on longer stairs was difficult due to increased pain and muscle weakness in the lower limbs as well as reduced balance. They described that they were afraid of falling when going up and down stairs and that they sometimes needed support from other people to manage. Some even said that they had to give up leisure activities, for example, swimming in the sea and meeting friends, because they could no longer manage the stairs.

*It can be a problem at the theatre or at other events, if you have to go up or down at these arenas [and manage the stairs]. You get scared, “what if I fall” and you get more unstable. In those moments it’s actually quite tough.* (p. 7)

*…we’re a group of friends who have known each other for many years. We’ve always met once a year [for dinner parties], but now I haven’t been there in three years. The host has stairs.* (p. 8)

#### 3.2.8. Travelling by Public Transport Was often Avoided Due to Fear of Falling and Physical Demands When Making a Journey

Travelling by public transport was perceived as challenging and, therefore, often avoided (cf. item 15). Some participants had experienced falls at the train station and were, therefore, afraid of travelling by public transport. They also told us that it could be difficult to find a seat on the bus or the train when it was crowded and to keep the balance when the bus was moving. Some had, therefore, been granted transportation service and no longer took the bus. Others described that they still managed to travel by public transport, if the bus or train were adapted to people with disabilities.

*I can’t take the bus, there’s rarely someone who gets up and offers a seat and when you get on the bus there are often a lot of people so it doesn’t work. I find it very difficult to keep balance when something’s moving under my feet.* (p. 2)

Travelling by air was also experienced as demanding according to some participants. It often included walking longer distances at the airport, as well as standing up for quite a while, which were perceived as very fatiguing.

*…the few times I’ve had to fly is nothing I’ve looked forward to. It’s far to walk and there are often long lines where you have to stand still and that’s tough.* (p. 5)

## 4. Discussion

To the best of our knowledge, this is the first study that has explored fall-related activity avoidance in persons with LEoP using a mixed-methods design. Our analysis revealed that many persons often avoided activities related to standing and walking, for example, taking a bath, performing household chores, walking outdoors, attending social events if there were stairs in the building and travelling by public transport, because of a fear of falling, increased pain and fatigability. To facilitate performance of daily activities, participants expressed that strategic thinking and various aids were important to use.

Ageing with LEoP often leads to a gradually increasing disability and, consequently, an increased risk of falling. It is previously described that falls may not only cause physical injuries, but also emotional and psychological distress, such as feeling embarrassed and a fear of becoming injured, which in turn, may lead to activity avoidance in daily life [16]. The occurrence of fall-related activity avoidance that our participants reported are very much in line with those reported by persons with Parkinson’s disease (PD) [18]. Kader et al. [18] described that the most common activities that persons with PD avoid are going out when it is slippery, reaching for something above their head and walking one kilometre. Our participants also commonly avoided going out when it was slippery and walking longer distances, but only sometimes avoided activities that required reaching for something above their head. Ageing with LEoP as well as PD affects balance and walking ability considerably, which may explain why activities requiring an ability to quickly adjust the body in case of a fall often are avoided. In persons with PD, it is described that a history of falls [27], greater fear of falling and disease severity [18] are associated with higher fall-related activity avoidance. Additionally, in older people, a greater number of falls, higher age and being a female are associated with increased activity avoidance due to fear of falling [17]. However, factors that are associated with fall-related activity avoidance in persons with LEoP remain unknown and need to be further explored in future studies.

Our participants described how their LEoP-related impairments and a fear of falling negatively affected their ability to perform many daily activities, especially outdoors. Their narratives revealed that they were vulnerable to barriers in different surroundings and to seasonal changes. Slippery or icy ground made it difficult to walk outdoors, which often forced them to be very cautious and, sometimes, to stay at home without any possibility to go out into the community. Moreover, being physically active, i.e., going for a walk with a friend, walking longer distances or climbing stairs, often caused increased muscle pain and fatigue. Additionally, travelling by public transport could be challenging, as it often meant standing for a long time and requiring an ability to quickly adjust the body to maintain the balance when the vehicle was moving. Hence, the participants perceived many challenges in daily life that forced them to be cautions, in line with previous studies in persons ageing with LEoP [5,16].

Even though some of our participants had developed problem-focused strategies and used mobility aids, anti-slip soles and acted carefully when walking, social participation became restricted in many situations. It has been previously shown [7,8,9] that falls often occur in activities related to walking, which may explain why such activities often were avoided. Additionally, in other qualitative studies of persons ageing with LEoP, the participants have described that walking limitations often limited them to perform daily activities [5,16] and to be physically active [28]. Thus, to address fall-related activity avoidance in relation to walking limitations is, therefore, important in the rehabilitation of persons with LEoP to increase functioning and to prevent a sedentary lifestyle.

Furthermore, our participants told us that indoor activities, requiring a high degree of balance and movement control, could be challenging because of their fear of falling. Small irregularities on the floor or on a carpet could pose a risk when walking indoors, and daily activities that were performed in a standing position, for example, showering or vacuuming, were often avoided. Standing up and preparing meals for longer times could also be difficult, but as many participants had developed problem-focused strategies, such as taking regular breaks and using various mobility aids, these activities were seldom avoided. Our findings are in line with previous studies of persons ageing with LEoP [5,16], describing that falls typically occur when taking a shower and standing on one leg to wash the feet, which may explain why many were afraid of performing this activity. Additionally, the increased impairments that many persons with LEoP perceive have meant that they must consider what activities they can do without experiencing too much pain, but also to develop strategies that work for them. It has previously been shown that persons ageing with LEoP, who had developed sufficient strategies, did not have to give up meaningful activities in daily life, which is important for their well-being [5]. Thus, development of functioning strategies is important to address in the rehabilitation of persons with LEoP.

Moreover, the participants in our study described that mobility aids and adaptations at home increased safety and facilitated some activities that could still be performed. Previous studies have reported that persons ageing with LEoP need more assistive devices over time to manage everyday activities [29] and that they feel more comfortable and safer when using mobility aids as they become older [5]. This might reflect an increased acceptance to one’s life situation when ageing with LEoP, and that persons over time have learned to live with their disability.

### 4.1. Clinical Implications

To avoid activities in hazardous situations can be considered as a sound strategy. However, if the fear of falling affects everyday life too much, social participation may become unnecessarily restricted. One way to reduce fall-related activity avoidance behaviour may be to participate in a fall management program, targeting a variety of physical and psychological aspects. To reduce the risk of falls, it may be important to diminish environmental barriers and to perform housing adaptations, but also to decrease LEoP-related impairments and to prescribe mobility aids. Previous studies in ambulatory persons with LEoP have shown that falls and/or fear of falling are associated with reduced walking ability, knee muscle strength [13,23] and dynamic balance [23]. Therefore, there are reasons to believe that interventions targeting these factors may potentially decrease falls and fear of falling. However, as experiences of falls could also lead to worries and distress [16] and altered behaviour, such as activity avoidance [17], interventions targeting self-efficacy (aiming to increase one’s confidence in the ability to dare to do things), learning how to find a balance between activities and rest and supporting the development of problem-focused strategies (in order to master skills and activities) may also be important to include in a fall management program. A previous study has shown that persons with LEoP can benefit from an individualized interdisciplinary rehabilitation targeting physical, psychological and environmental factors [30]. After rehabilitation, the participants experienced positive changes in their management of daily activities, in their view of their condition, their future and their self [30]. Thus, there are reasons to believe that persons with LEoP would also benefit from a fall management program, but further studies are warranted.

### 4.2. Strength and Limitations

A strength of this study was that the participants had experienced falls and fear of falling to a varying degree. By using a mixed-methods design and integrating data from mSAFFE and the interviews, a deeper understanding of the meaning of fall-related activity avoidance was achieved [20]. The participants provided rich data during the interviews, which gave valuable insights regarding how fall-related activity avoidance influenced their daily life. We included 14 persons with LEoP, and as no further information was added from the last interviews, we decided not to include any more participants. To maintain reflexivity [31], the authors had continuous discussions during the analysis to stay aware of our preunderstanding of persons with LEoP. To add transparency and trustworthiness to our findings, we presented the participant’s identification code after each quotation. There were also some limitations. No participants expressed that they avoided going to the doctor or dentist during the interviews, and therefore, this aspect was not highlighted in the qualitative analysis. Even though mSAFFE has shown good psychometric properties in older people [17] and in persons with PD [32], its validity and reliability needs also to be evaluated in persons with LEoP. Additionally, factors associated with fall-related activity avoidance in persons with LEoP need to be studied.

## 5. Conclusions

Fall-related activity avoidance is common in persons with LEoP, which negatively influence daily life and social participation. To increase daily functioning in this population, fall-related activity avoidance should be included in a multifaceted fall management program.

## Figures and Tables

**Table 1:** Demographics and characteristics of the 14 participants with late effects of polio.

**Age**	
Mean years (SD), range	70 (5), 61–78
**Age at acute polio**	
Mean years (SD), range	4 (4), 1–12
**Age at onset of LEoP**	
Mean years (SD), range	48 (8), 37–64
**Duration of new LEoP related impairments**	
Mean years (SD), range	26 (9), 9–43
**Self-perceived degree of LEoP**	
Mild/moderate/severe (n)	3/6/5
**Living situation**	
Living with a partner/alone (n)	11/3
**Resident**	
Living in a house/apartment (n)	8/6
**Ground around the residence**	
Even/uneven (n)	12/2
**Walking ability (n)**	
<100 m/ between 100 and 1000 m/ > 1000 m	3/7/4
**Using mobility aids**	
Indoors or outdoors (n)	11
**Fall frequency the past year**	
0 time/1–3 times/4–6 times	2/7/5
**Fear of falling ^a^**	
Mean (SD), range	33 (11), 20–55

SD = standard deviation. LEoP = late effects of polio; ^a^ = assessed by the Falls Efficacy Scale—International. Score ranges between 16 and 64 points; higher scores indicate more concerns about falling.

**Table 2:** Occurrence of fall-related activity avoidance in persons with late effects of polio.

mSAFFE, Item	Never n (%)	Sometimes n (%)	Always n (%)
**1.** Walk to the store and shop	6 (43)	7 (50)	1 (7)
**2.** Clean your house	8 (57)	2 (14)	4 (29)
**3.** Prepare simple meals	12 (86)	2 (14)	0
**4.** Go to the doctor or dentist	12 (86)	2 (14)	0
**5.** Take a bath	5 (36)	7 (50)	2 (14)
**6.** Take a shower	9 (64)	4 (29)	1 (7)
**7.** Go for a walk	2 (14)	10 (71)	2 (14)
**8.** Go out when it is slippery	0	6 (43)	8 (57)
**9.** Visit a friend or relative	10 (71)	3 (21)	1 (7)
**10.** Walk to a place with crowds	5 (36)	8 (57)	1 (7)
**11.** Climb stairs	3 (21)	8 (57)	3 (21)
**12.** Walk around indoors	12 (86)	2 (14)	0
**13.** Walk a kilometre	1 (7)	7 (50)	6 (43)
**14.** Bend down to pick up something	10 (71)	4 (29)	0
**15.** Travel by public transport	5 (36)	5 (36)	4 (29)
**16.** Attend a social event or party	9 (64)	5 (36)	0
**17.** Reach for something above your head	7 (50)	6 (43)	1 (7)

mSAFFE = modified Survey of Activities and Fear of Falling in the Elderly.

**Table 3:** Categories that emerged from the deductive analysis and their relation to the items in mSAFFE.

Category	Item
Going out when it was slippery was often avoided due to fear of falling	8
Challenging to walk longer distances because of increased LEoP-related impairments	7, 13
Mobility aids facilitated going to the store and other crowded places	1, 10
Walking indoors was rarely avoided but could still be very challenging	12
Household chores could be physically demanding and cause strain on the body	2, 3, 14, 17
Taking a shower was seldom avoided but was carried out with precautionary measures	5, 6
Attending social events could be problematic if there were stairs in the building	9, 11, 16
Travelling by public transport was often avoided due to fear of falling and physical demands when making a journey	15

## Data Availability

Data are available only upon request to the authors, according to the ethical approval from the Swedish Ethical Authority.
